# Inaccessible and Deceptive: Examining Experiences of Deceptive Design with People Who Use Visual Accessibility Technology

**DOI:** 10.1145/3706598.3713784

**Published:** 2025

**Authors:** Aaleyah Lewis, Jesse J Martinez, Maitraye Das, James Fogarty

**Affiliations:** University of Washington, Seattle, Washington, USA; University of Washington, Seattle, Washington, USA; Northeastern University, Boston, Massachusetts, USA; University of Washington, Seattle, Washington, USA

**Keywords:** accessibility, deceptive design, dark patterns, visual accessibility technology, screen readers

## Abstract

Deceptive design patterns manipulate people into actions to which they would otherwise object. Despite growing research on deceptive design patterns, limited research examines their interplay with accessibility and visual accessibility technology (e.g., screen readers, screen magnification, braille displays). We present an interview and diary study with 16 people who use visual accessibility technology to better understand experiences with accessibility and deceptive design. We report participant experiences with six deceptive design patterns, including designs that are intentionally deceptive and designs where participants describe accessibility barriers unintentionally manifesting as deceptive, together with direct and indirect consequences of deceptive patterns. We discuss intent versus impact in accessibility and deceptive design, how access barriers exacerbate harms of deceptive design patterns, and impacts of deceptive design from a perspective of consequence-based accessibility. We propose that accessibility tools could help address deceptive design patterns by offering higher-level feedback to well-intentioned designers.

## 1 Introduction

Recent work has focused attention on deceptive design patterns (also known as “dark patterns”)1 [14], interface designs that manipulate people into performing actions to which they would otherwise object [57]. For example, a service that allows easy subscription, but then makes unsubscribing difficult, employs an ‘Obstruction’ pattern to manipulate people into continuing a subscription [35, 57]. Importantly, contemporary definitions of deceptive design focus on the impacts of design choices or design inaction, without requiring that a designer intentionally aim to deceive [16, 26]. Prior theoretical and empirical research on deceptive design has largely focused on development of taxonomies to characterize patterns [11, 20, 35, 39, 52, 57]. Such work has identified deceptive design patterns at scale in e-commerce [57], voice assistants [59, 67], social media [50, 60, 62], mobile applications [25], gaming [24, 69, 88], safety technology [18], privacy [11, 28, 32, 37, 65, 77], and proxemic interactions [39]. Alongside these, work has examined end-user perception of deceptive design patterns [9, 25, 33, 52, 54, 59, 60].

Given the prevalence of deceptive design, we argue it is crucial to better understand how these patterns impact people who use visual accessibility technology (e.g., screen readers, screen magnification, braille displays), as the difference in interaction modality can create different manifestations and impacts of these patterns. Prior accessibility work has explored web and mobile accessibility barriers [17, 40–42, 61, 72, 76] and examined the impact of these barriers on people who use visual accessibility technology [31, 48, 49]. Further, research has explored workarounds devised to navigate accessibility barriers [5, 10, 31, 48, 51, 63, 83]. Despite such work on web and mobile accessibility, there remains limited exploration of the interplay of accessibility and deceptive design.

Our research aims to better articulate the interplay between accessibility and deceptive design, including further characterizing the impacts of deceptive design patterns encountered by people using visual accessibility technology. We broadly consider online services that provide varying information and resources (e.g., banking, blogs, entertainment, news, shopping, social media, travel), and we examine deceptive design patterns through a lens of accessibility to identify exacerbated challenges and consequences. We report findings from semi-structured interviews and a subsequent diary study with 16 disabled people who use visual accessibility technology, focusing on two research questions:

- **RQ1:** What deceptive design patterns do people who use visual accessibility technology encounter in online services?
- **RQ2:** How do accessibility barriers interplay with deceptive design patterns to impact people who use visual accessibility technology?

Our analysis reveals complexities in the interplay of accessibility and deceptive design. Participants describe differential and amplified forms of manipulation, participants describe how they felt accessibility barriers manifest as deceptive design patterns, and participant experiences reveal disproportionate direct and indirect consequences.

This paper makes four primary contributions:

- We present an interview and subsequent diary study with 16 participants who use visual accessibility technology, examining their experiences with deceptive design patterns and their interplay with accessibility.
- Informed by existing taxonomies of deceptive design [35, 57], we report six deceptive design patterns participants described encountering in online services when using visual accessibility technology: ‘Sneaking’, ‘Forced Action’, ‘Misdirection’, ‘Obstruction’, ‘Urgency’, and ‘Nagging’. Participant experiences included both accessibility barriers amplifying challenges of deceptive design and accessibility barriers they felt manifested as deceptive designs.
- We report participant accounts of harms in the interplay of accessibility and these deceptive design patterns, including direct harms of intentional deceptive designs, similar harms due to accessibility barriers manifesting as deceptive design patterns, and indirect harms of participant workarounds.
- We discuss implications of participant experiences and insights in the interplay of accessibility and deceptive design, specifically considering: intent versus impact of deceptive design patterns, harms of deceptive design patterns for people who use visual accessibility technology, deceptive design patterns and tradeoffs through a lense of consequence-based accessibility, and design considerations for accessibility tools.

## 2 Related Work

Our study is informed by prior research in deceptive design patterns and prior research in web accessibility.

### 2.1 Deceptive Design Patterns

#### 2.1.1 Understanding Deceptive Design Patterns.

Deceptive design patterns use cognitive biases and heuristics to undermine agency through stylistic choices, emotion, and language [57]. This exploitation largely relies on understanding human decision-making processes [79], a strategy commonly used in manipulation [79], persuasive design [27], and nudging [80, 87]. In HCI and CSCW contexts, Susser et al. define manipulation as the “hidden influence”, influencing a person’s decision-making by exploiting their decision-making vulnerabilities [79]. Manipulation influences the way people perceive available actions [79], restricting their freedom of choice [68].

A growing body of research has focused on the persuasive and manipulative practices of deceptive design patterns, including in e-commerce [57], voice assistants [59, 67], social media [50, 60, 62], mobile applications [25], gaming [24, 69, 88], safety technology [18], privacy [11, 28, 32, 37, 65, 77], proxemic interactions [39] and the ethical complexities of these practices [34, 75]. Work has also examined end-user perspectives on the impacts of deceptive design patterns [9, 25, 33, 52, 54, 59, 60]. Maier and Harr conducted interviews to examine experiences with deceptive design patterns, finding participants experience annoyance and frustration while also feeling partially responsible for their own manipulation [54]. Gray et al. conducted a survey examining emotional impacts in experiences of exploitation and providing insights into end-user perceived responsibility for manipulation [33]. Bhoot et al. examined factors that impact end-user reactions to deceptive design patterns, presenting a set of tasks and exploring participant recognition of and reactions to deceptive design in terms of variables including frustration, trustworthiness, and frequency of occurrence [52]. Bongard et al. conducted surveys to understand end-user awareness of the influence of manipulative designs on their actions. Despite reported awareness, participants lacked necessary skills to oppose deceptive designs [9]. Similarly, Mildner et al. assessed expert ability to identify deceptive patterns in social networking services [60]. Geronimo et al.’s survey experiment found most people are not able to identify malicious designs in mobile apps, emphasizing the need to increase awareness [25]. To explore ethical caveats and unethical practices in conversational interfaces, Mildner et al. interviewed researchers, practitioners, and frequent users of conversational interfaces, finding experiences of frustration, mistrust, and misaligned expectations [59].

#### 2.1.2 Deceptive Design Pattern Taxonomies.

Many existing taxonomies of deceptive design patterns build upon Brignull’s original work [14]. Additional work often aims to provide further understanding through theoretical insights and empirical studies, and Gray et al. recently proposed an ontology and corresponding language for categorizing deceptive design patterns [36, 38]. We most directly build upon a taxonomy of seven categories of deceptive design patterns developed by Mathur et al. [57]. Informed by Brignull [14] and Gray et al. [35], Mathur et al. examined 11K shopping websites to identify and develop a characterization of the cognitive influences of deceptive design patterns. Mathur et al.’s seven resulting categories of deceptive design patterns are defined as: *‘Sneaking’* misrepresents available actions by hiding or delaying information; *‘Urgency’* imposes deadlines or time constraints to accelerate decision-making and completion of an action; *‘Misdirection’* uses visuals, language, and emotion to steer people toward or away from certain decisions; *‘Social Proof’* presents the actions of others to exploit a bandwagon effect in decision-making; *‘Scarcity’* increases perceived value by suggesting limited availability or high demand; *‘Obstruction’* makes certain actions harder than they should be in order to dissuade people; and *‘Forced Action’* requires additional and tangential actions in order to complete a desired task. Mathur et al. also characterize how deceptive design patterns decrease wellbeing: diminishing it through financial loss, invasion of privacy, and cognitive burdens [58]. Our work directly leverages Mathur et al.’s categories of deceptive design patterns for exploring participant experiences with accessibility and deceptive design, while we also contribute new understanding of experiences and consequences in the interplay of accessibility and these patterns.

### 2.2 Web Accessibility and Usability for People Who Use Visual Accessibility Technology

Researchers and practitioners have developed guidelines aimed at ensuring people with disabilities can “perceive, understand, navigate, and interact with the Web” [84]. The most popular standard is the World Wide Web Consortium (W3C)’s Web Content Accessibility Guidelines (WCAG), first introduced in 1999 [85]. Among such guidelines [85, 86], examples for people who use visual accessibility technology include: appropriate alternative text for images; adding proper labels to elements (e.g., buttons, input form) so screen readers can announce their functionality; enabling proper heading levels to better support keyboard navigation; and incorporating skip navigation links to support directly accessing primary content.

Despite such guidelines and best practices being well established, accessibility barriers continue to persist. A recent survey of 1 million home pages detected an average of 50 errors and WCAG conformance failures per page [81]. Moreover, people who use visual accessibility technology experience many usability problems not adequately captured by official standards [6, 8, 10, 40, 51, 70, 72, 82, 83]. For example, Borodin et al. [10] report that people find it difficult to extract required information when sifting through web content that is being dynamically updated. For people who use screen readers, the severity of this issue sometimes reaches a point where they may fail to identify if the information they seek is inaccessible or altogether absent, a challenge Bigham et al. [6] termed “not knowing what you don’t know.” Researchers have sought to address such accessibility issues through development of assistive technology incorporating shared browsing [66] and semantic web modeling techniques [2, 3, 7]. Others have proposed techniques for faster non-visual skimming of web content using browsing history to infer individual preferences and personalize reading adaptations [2, 15, 29]. Collectively, this body of work uncovers and addresses several core issues associated with accessing web content using visual accessibility technology. However, relatively little is known about how these accessibility issues interplay with deceptive design patterns to impact web experiences for people who use visual accessibility technology.

### 2.3 Understanding the Interplay of Accessibility and Deceptive Design

Although substantial prior research has examined both accessibility and deceptive design, such work has largely been separate and thus not characterized challenges in their interplay. A recent notable exception is research by Kodandaram et al. [47]. They conducted interviews with 18 blind participants, examining how deceptive design patterns in web advertisements impact people who use screen-readers, finding that blind people who use screen readers are often misled by contextually integrated ads and encounter challenges using ad blockers. They developed an algorithm to improve automatic identification of deceptively integrated ads (e.g., ads embedded within search results). Our work explores a much broader range of participant experiences with deception, using Mathur et al.’s taxonomy of deceptive design patterns to elicit participant experiences with accessibility across many forms of deception. We also find that participants describe experiencing accessibility barriers as deceptive, regardless of designer intent, and we report participant experiences with direct and indirect harms of these deceptive design patterns.

Adjacent research has explored the interplay of accessibility and challenges in privacy and security. For example, Clarke et al. investigated accessibility challenges of website cookie notices for people with visual impairments, combining evaluations of 46 UK websites with a survey study with 100 visually impaired people [19]. Their findings reveal insights into a disconnect between compliance with accessibility guidelines and data protection regulations versus experiences with those notices (e.g., despite meeting compliance criteria, many notices present significant barriers in privacy management for visually impaired people). Janeiro et al. conducted an interview study and lab sessions with people who use screen readers to understand their experiences encountering phishing, their defenses to phishing, and the inaccessibility of phishing countermeasures [45]. Gaggi et al. conducted a survey study with people with visual impairments to examine the accessibility of Google reCAPTCHA systems, finding reCAPTCHA v2 presents accessibility barriers for people with visual impairments whereas reCAPTCHA v3 is more accessible [30]. Such work motivates examining how accessibility interplays with challenges in privacy and security, which we explore in our focus on deceptive design patterns.

## 3 Methods

### 3.1 Participants

We conducted semi-structured interviews with 16 people who primarily access online services using visual accessibility technology (i.e., screen readers, screen magnification, braille displays, voice assistants). Although our work primarily captures the experiences of blind and low-vision participants, we interviewed one participant who self-described as chronically ill and uses a screen reader to mitigate motion sensitivity. We recruited participants through multiple channels: posts to public social media, posts to a local disability-focused research community, and a research participation solicitation distributed by the National Federation of the Blind. Our specific inclusion criteria included: (1) at least 18 years old; (2) have a disability or chronic condition; (3) use accessibility technology when navigating online services. Of 16 participants, 15 reported primarily using screen readers (e.g., Fusion, JAWS, NVDA, Talkback, Voiceover) and 1 reported primarily using voice assistants. In addition, some participants reported using braille displays, screen magnification, and voice assistants. Participants reported between 1 and 35+ years of experience with their accessibility technologies. Table 1 provides additional self-reported details of participants.

Throughout our analysis, we keep in mind that each participant’s experiences are different (e.g., due to varying access needs, varying expertise with accessibility technologies, sociocultural factors impacting accessibility technology usage and adoption [46, 55, 71, 74]). Although our analysis primarily focuses on accessibility and deceptive design as experienced using screen readers, some participants describe experiences with screen magnification, braille displays, and voice assistants. We therefore use the term ‘visual accessibility technology’ to keep our language true to their reported experiences, and additional examination of how other accessibility technologies shape such challenges remains an important area of future research.

### 3.2 Procedure

Semi-structured interviews each lasted approximately 1 hour, including four phases:

1. We asked participants to self-report their disability, their accessibility technology background, their preferred technology configuration, and the types of online services they commonly use.
2. We asked participants to discuss accessibility barriers they commonly encounter in online services and how those impact their decision-making and engagement. We then asked participants to discuss workarounds and strategies they use to manage these accessibility barriers. This primarily provided context for us to better understand and convey each participant’s accessibility experiences throughout the rest of the interview.
3. To scaffold participant exploration of their experiences, we introduced the overall concept of deceptive design patterns, then each of Mathur et al.’s seven categories of deceptive design [57] (i.e., in the order ‘Sneaking’, ‘Forced Action’, ‘Misdirection’, ‘Obstruction’, ‘Scarcity’, ‘Urgency’, ‘Social Proof’). After introducing each pattern, we asked participants to reflect on experiences encountering that pattern. We then asked participants to share any difficulties they had navigating the pattern using their visual accessibility technology, and any established workarounds to help manage the pattern. This aimed to capture how deceptive patterns are amplified by accessibility barriers.
4. We asked participants to reflect on the set of deceptive design patterns and to discuss anything they felt was missing or could better describe their experiences. In contrast to the previous phase’s focus on participant experiences with well-established patterns, this aimed to elicit experiences of people with disabilities that may not be captured in existing understandings of deceptive design.

All interviews were conducted by the first author. Prior to each interview, we sent the participant a study information and consent sheet and a document detailing topics we would explore in the interview. This gave participants an option to review and prepare talking points beforehand. Interviews were conducted and recorded using Zoom, except for one conducted by phone using Rev Call Recorder per the participant’s request. Our university’s Institutional Review Board reviewed and approved this research, and participants were compensated with a $30 gift card for their time.

Upon completion of the interview, we gave each participant the option to participate in a two week follow-up diary study to share additional in-context experiences encountering deceptive design patterns. In addition to providing another path for participants to share, we intended the diary study to support participants who had difficulty recalling past experiences during the interview or who found themselves more aware of deceptive design patterns after the interview. For each diary entry, we asked participants to document: (1) what online service they were using, (2) what action they were attempting to complete, (3) what challenges they encountered in performing this action, and (4) any workarounds they employed. Apart from presenting the diary study as an additional opportunity to share, participants were not given any specific guidance on what experiences to share (i.e., the diary study did not aim to capture all experiences nor any specific subset of experiences). Each report was compensated with an additional $5, up to a maximum of $30 in diary study compensation. Of 16 interview participants, 11 (P3, P4, P5, P6, P8, P9, P10, P12, P13, P15, P16) participated in the diary study providing a total of 24 responses.

### 3.3 Data Analysis and Reporting

We transcribed interviews using secure audio transcription software. We analyzed interview and diary data together, using a codebook thematic analysis approach [13]. We applied a combined deductive-inductive approach to coding, drawing between broader theoretical frameworks and participant reported experiences. Deductive codes were derived from taxonomies created by Mathur et al. [57, 58] and Gray et al. [35, 36]. Additional inductive codes were developed by the first author by reviewing and open-coding 16 interview transcripts for topics related to participant experiences with deceptive design patterns. Codes were discussed and revised as a group among the first three authors to produce an initial codebook which then guided coding of all interview transcripts and diary entries. The first author coded all interview transcripts, and the second and third authors individually coded separate subsets to bring alternative perspectives to the data. The first three authors discussed codes throughout the coding process to resolve conflicts and to develop initial themes. In parallel, the first author coded all diary entries while the third author reviewed a subset for consistency. Also in parallel, the first author reviewed online services in which participants reported encountering deceptive design patterns (i.e., drawing on data from both interviews and diary entries), both to mitigate potential recall bias and to support interpretation of participant descriptions of their experiences. We finally organized our findings into high-level themes that capture: (1) how participants described encountering deceptive design patterns in online services and (2) the impact of those experiences.

**Figure 1:**
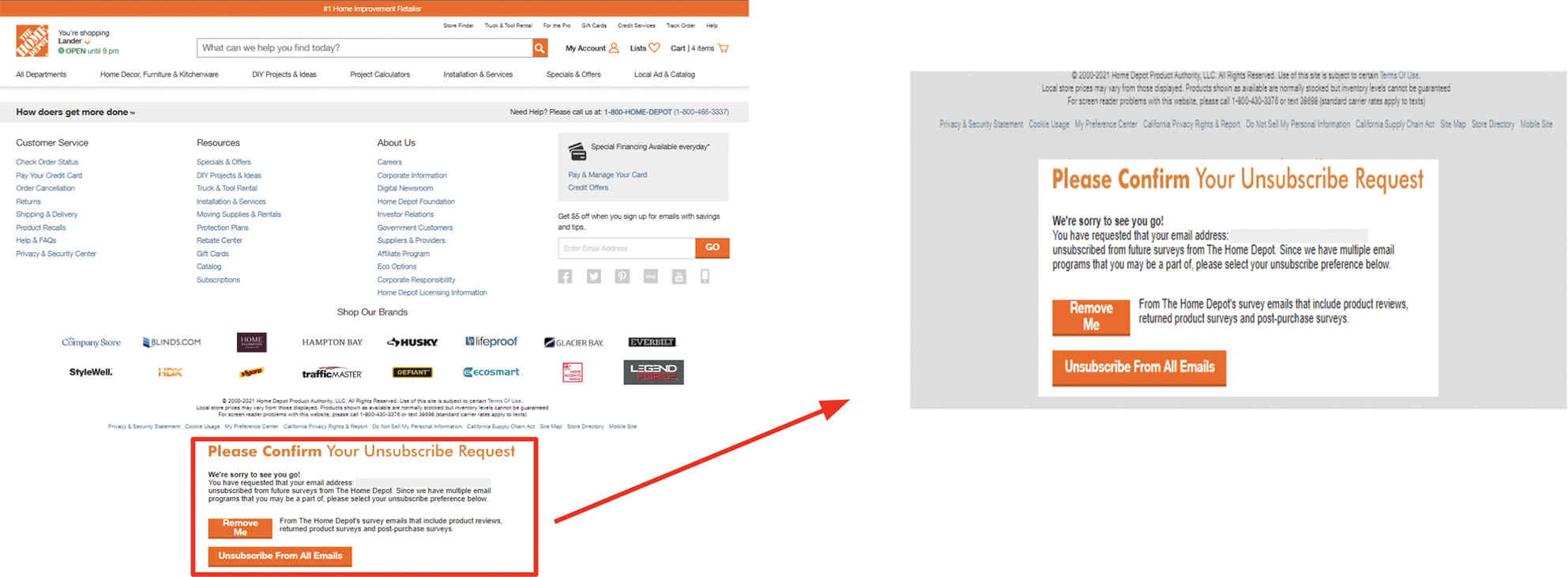
In a diary entry, P4 described an ‘Obstruction’ pattern manifested in using screen magnification on HomeDepot.com. The unsubscribe link was at the bottom of the page, such that P4 described it as taking longer to find using keyboard navigation.

In reporting examples throughout this paper, we explicitly note whenever a reported example was provided through a diary entry (e.g., Figure 1 notes P4’s description of an ‘Obstruction’ pattern was shared in a diary entry). If not explicitly stated as a diary entry, reported examples were shared in an interview.

### 3.4 Positionality

Our analysis and writing are informed by our experiences and identities. Our research team is composed of scholars with and without disabilities, graduate students, and senior academic faculty. The authors have experience conducting accessibility and human-computer interaction research. We acknowledge that our scope of disability and accessibility is contextualized to the United States based on our collective positionality and experiences.

## 4 Results

Participants shared examples of using services across 11 categories: banking, blogs, education, email, entertainment, medical, news, shopping, social media, travel, and weather. Participants described experiences with the interplay of accessibility and five of Mathur et al.’s seven categories of deceptive design patterns [57], specifically ‘Sneaking’, ‘Forced Action’, ‘Misdirection’, ‘Obstruction’, and ‘Urgency’. Although they were not explicitly introduced to the concept, participants also described experiences of Gray et al.’s ‘Nagging’ pattern, defined as a redirection of expected functionality that may persist over one or more interactions [35]. Participants were explicitly introduced to all of Mathur et al.’s categories, but did not describe accessibility-related experiences relative to ‘Scarcity’ or ‘Social Proof’.

Our original expectation was to elicit additional challenges of accessibility in *intentional* examples of deceptive design patterns. However, participants also described examples where they felt an accessibility barrier (e.g., an unlabeled element) resulted in a design that was deceptive only when using visual accessibility technology (i.e., might not be experienced as deceptive by people not using visual accessibility technology). An accessibility barrier can therefore manifest as a deceptive design pattern that is likely *unintentional*. Participants discussing their experiences and the impacts of deceptive design patterns often did not distinguish designer intent in specific examples of those patterns, and intent can sometimes be difficult to assert (e.g., a designer may implement a pattern without realizing it is deceptive). To stay consistent with participant descriptions of their own experiences, this section emphasizes participant description of experiences with key properties of deceptive design patterns, providing specific participant-reported examples as context for understanding more general experiences with those patterns. Section 5’s discussion then revisits consideration of intent versus impact in accessibility and deceptive design.

This section reports participant experiences with deceptive design patterns, additional experiences with the interplay of multiple deceptive design patterns, and then participant description of the impact of these experiences.

### 4.1 Experiences with Visual Accessibility Technologies and Deceptive Design Patterns

Table 2 summarizes six deceptive design patterns participants described encountering using visual accessibility technology across a variety of online services. Participant experiences intersect with related access barriers that are widely discussed in prior research (e.g., unlabeled and uninformative elements [17, 41, 42, 61, 76]). We thus focus on and contribute an examination of the interplay between these established access barriers and deceptive design patterns. This includes deceptive design patterns that are amplified by access barriers (e.g., ‘Sneaking’, ‘Obstruction’, ‘Nagging’ patterns amplified by unlabeled elements and problematic keyboard navigation order), thus causing disproportionate harms to people who use visual accessibility technology. It also includes familiar access barriers (e.g., unlabeled elements) that participants described manifesting as a deceptive design pattern, thus causing greater harms than might otherwise be associated with that barrier. Because this interplay between access barriers and deceptive design manifests differently across different contexts, we separately report participant experiences with each pattern. We report patterns in the same order they were presented to participants, concluding with the inductively-identified ‘Nagging’.

#### 4.1.1 Sneaking.

‘Sneaking’ refers to designs that misrepresent actions by hiding or delaying information that would likely lead people to object [35, 57]. Participants shared examples of ‘Sneaking’ due to missing or uninformative labels for interface elements (e.g., buttons, checkboxes, sliders). All participants reported encountering elements that screen readers announced only as “button”, “unlabeled button”, or nothing at all when attempting tasks such as removing items from a shopping cart. In a diary entry, P10 described making a wish list on Amazon, during which they experienced an undesired dialog overlaying the main window, *“which in turn made it actually easier to add to cart rather than my wish list”*, exemplifying the ‘Sneaking into Basket’ pattern [57]. Participants described other experiences where overlaid windows or absence of labels effectively disguised, hid, or misrepresented information available through visual accessibility technology and led to undesired actions. Several described unintentionally subscribing to promotions because they were unable to identify unlabeled pre-selected fields (e.g., checkboxes). P15 said, *“During my physical therapy experience, it either won’t say at all when something’s checked or I’ll try to uncheck something and it won’t be clear whether it’s unchecked.”* P4 also commented, *“If your screen reader doesn’t focus on it, great! You’ve been subscribed to more spam.”* P1 described, *“It might be harder for me to locate those fields and fill them in and check what’s mandatory and what’s not as a screen reader user versus when somebody is just looking at it.”* Such instances of ‘Sneaking’ become more challenging in the context of monetary transactions. For example, P14 discussed increased risk of unlabeled elements in financial services, *“I don’t know what the information is that’s trying to be conveyed here. But obviously if it’s a banking app, that’s important to me.”* Participants described exercising greater caution in such situations, as P15 noted, *“I do have to make sure that I really listen and see whether something is checked or not... if it’s something important, like a protection plan or something extra that I have to pay for.”*

#### 4.1.2 Forced Action.

‘Forced Action’ refers to designs that require additional tangential actions to continue in a desired task [35, 57]. Participants described ‘Forced Action’ when services require authentication using inaccessible visual and audio options (e.g., reCAPTCHA v2). Although these are imposed on everybody using a service, participants described them as deceptive because accessibility barriers create additional challenges in completing intended tasks [44]. For instance, reCAPTCHA v2 uses ‘I am not a robot’ checkboxes, static image recognition, or distorted text and number recognition tasks to distinguish people from robots. The vision-centric nature of these CAPTCHAs inherently exclude many blind and low-vision people [30, 44]. Although reCAPTCHA v2 includes an alternative audio configuration, this still creates barriers due to difficulty of the task. P7 explained, *“The reason CAPTCHA is evil is that if there’s an audio option, it’s often muffled and there’s noise in the background to try to discourage bots, which also discourages me.”* P6 added, *“Sometimes the audio CAPTCHA would be so distorted it would be hard to understand, and I would have to hear it several times before I could figure out what the audio CAPTCHA is.”* CAPTCHAs thus prevented participants from proceeding with their intended tasks and instead forced spending time and effort on inaccessible authentication tasks. Participants understood the necessity of such security, but felt that deploying them without adequate accommodations for people with disabilities effectively denied them their rights to access.

#### 4.1.3 Misdirection.

‘Misdirection’ refers to designs that use presentation, language, and emotion to steer people toward or away from certain decisions [57]. Prominent examples described by participants involved elements with inaccessible stylistic choices. Low-vision participants described heightened challenges with inaccessible stylistic choices (e.g., low color contrast, illegible typography, small font size). P12 described buttons with low color contrast such that people are coerced into signing up for services: *“Sign up button is red with white writing and then under that, in light blue on a white background, it’s going to say ‘No thanks’.”* P4 shared feeling manipulated [33] through such ‘Misdirection’: *“I feel like oftentimes the ones that they don’t want you to pick are grayed out more and [are] therefore lower contrast.”* P12 reported attempting to message their Uber driver but being misdirected to cancel the ride because *“the app changed the location of the cancel button and made it the same color and shape of the message button. It also just said ‘button’ with VoiceOver”* (i.e., as an unlabeled element). P13 shared an example on a dental website where mislabeled elements created confusion: *“There’s a submit button and a cancel button. They’re both labeled submit... So every once in a while it’ll trip me up.”*

#### 4.1.4 Obstruction.

‘Obstruction’ refers to designs that make actions needlessly difficult to dissuade those actions [35, 57]. P7 described ‘Obstruction’ on the Delta Airlines website, where unlabeled elements made it *“almost impossible”* for them to change a flight. Participants often described ‘Obstruction’ due to unlabeled or mislabeled elements that made it difficult to detect an unsubscribe option. In a diary entry, P10 said, *“In the [subscription] email, there is not a clearly labeled unsubscribe link. I went to the website to find an unsubscribe section, I was unable to find it.”* Participants observed that unsubscribe links sometimes redirected them to a new page with its own accessibility barriers. P10 described, *“There’s been definite times you can’t find the link to unsubscribe or once you do, then the webpage to unsubscribe is not accessible.”* In other cases, participants described unsubscribe links located far down a page such that it would take longer to find when using visual accessibility technology. In a diary entry, P4 reported this in an email from Home Depot: *“The page put the unsubscribe info ALL the way at the bottom of the page such that I had to scroll to find it with average magnification in my browser, which did take me a minute. The site also put that information way down in the tab order, so you’d have to [repeatedly] tab down to get to it if using a screen reader.”* (see Figure 1).

Participants similarly described dismissal buttons on disruptive ads (e.g., ‘X’, ‘Close’, ‘No thanks’) were *“often not labeled”* (P5, P8, P10, P13, P14) or could not be activated with default keystrokes, which made ads *“hard to close”* (P13). While trying to read a news article, P10 was unable to dismiss a recurring subscription ad, *“I repeatedly tried to navigate to the close button and attempted to close the pop-up, refreshing the screen a few times between attempts... But the close button was hard to find and not responsive.”* P8 shared, *“If I can’t find a close button, I will hit either ‘Alt F4’ or ‘Control F4’, thinking I’m closing a tab and it will instead close out the entire website.”* ‘Obstruction’ of P8’s intended action (i.e., closing an ad) thus led to unintended action (i.e., closing an entire site). Participants further described challenges with delayed dismissal options (e.g., that appear after other elements of an advertisement). P10 shared when they *“passed over [an unlabeled advertisement] and then if you were to go backwards through the text or whatever, then all of a sudden it’s labeled. Especially with the close button on pop-up ads and stuff, that tends to be a thing I see a lot.”* Participants further noted these barriers can be particularly disruptive in combination, as with a delayed dismissal option that is then also not properly labeled.

#### 4.1.5 Urgency.

‘Urgency’ refers to designs that impose deadlines or time constraints to accelerate decision-making and action [57]. Participants described ‘Urgency’ in services that used countdown timers to accelerate decision-making. For example, P7 encountered a countdown timer in a ticketing website and felt they *“better really decide if I want this because it’s only going to last another ten minutes.”* Participants further described some countdowns being continuously announced by their screen reader, becoming *“very disruptive”* (P13). P8 explained, *“Those can be very distracting when you’re trying to pay for something or trying to do whatever. You’ve got 15 minutes to make a purchase and you keep hearing that countdown in the background and you’re trying to get past that.”* To work around this, P8 described sometimes switching to their braille display, however, *“if [the braille display] does the same thing — if it’s counting down on the braille display — then the only thing I can do is get sighted assistance because they can bypass that a little easier.”* Despite this challenge, P7 said that screen readers not announcing the presence of countdown timers at all could also create a feeling of missing out, and other participants commented that countdown timers could be helpful if the remaining time was announced in intervals.

#### 4.1.6 Nagging.

**Figure 2:**
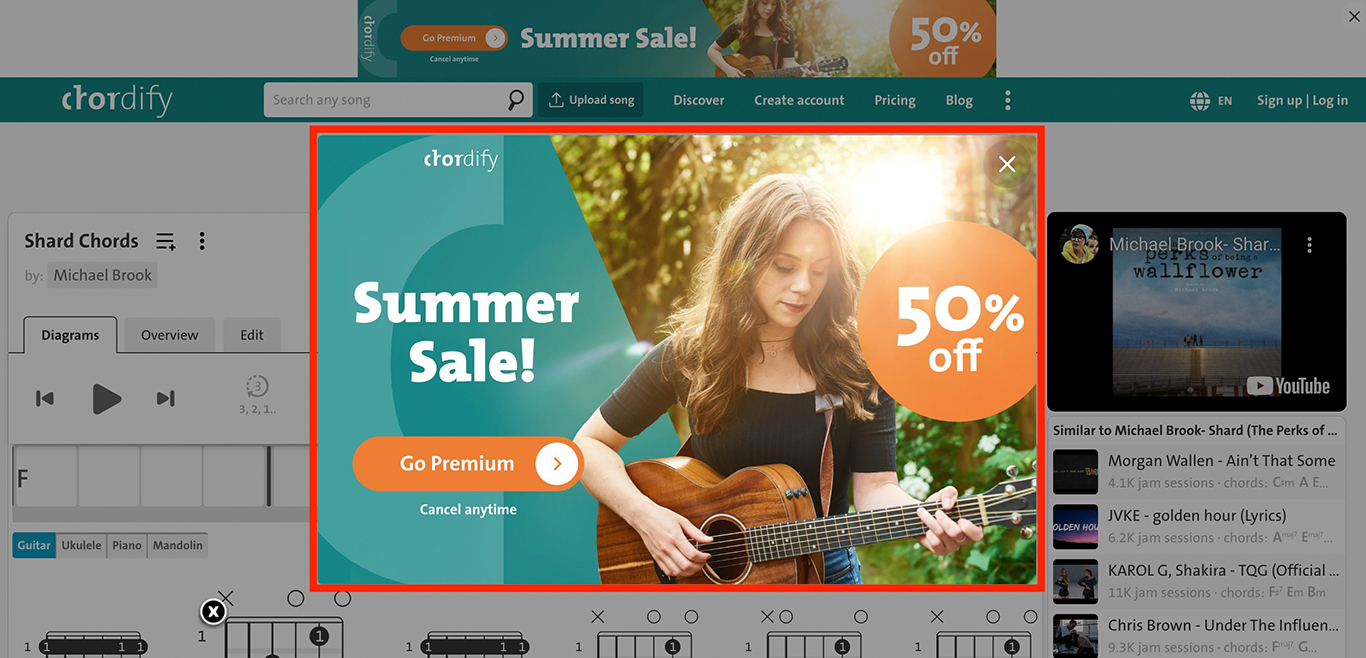
P14 described a ‘Nagging’ pattern on Chordify.net amplified by accessibility barriers manifested in their use of a screen reader. While using this website to learn music chords, a pop-up ad caused repeated intrusion after every completion of a song. P14 could not easily dismiss the ad, and they instead described a workaround of refreshing the page and starting over.

**Figure 3:**
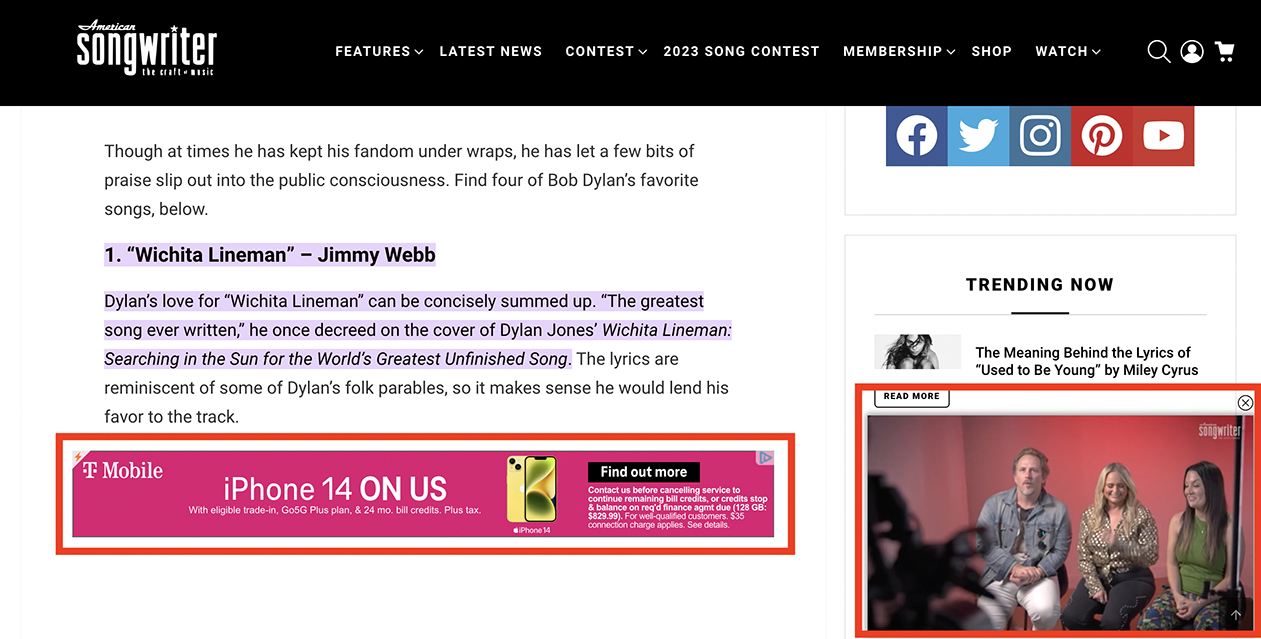
In a diary entry, P13 described a ‘Nagging’ pattern on AmericanSongwriter.com exacerbated by accessibility barriers manifested in their use of a screen reader. While reading an article, P13 described repeated intrusion by an automatically-played audio-video ad and recurring banner ads, preventing P13 from completing the article.

‘Nagging’ refers to designs that impose repeated intrusions of unexpected functionality unrelated to a person’s task [35]. Participants most commonly described ‘Nagging’ through repetitive advertisements in pop-up overlays. Although annoying for everyone, participants described how accessibility barriers make these substantially more disruptive for people who use visual accessibility technology. For example, P14 shared how recurring advertisements continued to interrupt them on a music chords website, *“Every time I finish looking at the song with the chords, it pops up with this ad... So I always just refresh the page and I have to start the song over. It is very difficult to navigate with my screen reader.”* (see Figure 2). P12 recalled ‘Nagging’ ads on Reddit, *“If you’re reading a post and then you want to read the comments, if there’s an ad there, it’ll just stop at the ad and won’t go to the comments. It’s really annoying when those ads [come up].”* In a diary entry, P13 described reading a blog post linked from Facebook, *“An ad kept coming up and as I would try to scroll to continue reading, my phone would completely freeze on the ad and I never was able to finish the article.”* (see Figure 3).

Participants further described ads moving their screen reader focus away from areas of interest, making it challenging to access intended information. This was sometimes due to ads placed higher in the keyboard navigation order (i.e., the order in which a screen reader moves through elements). P16 explained, *“It is annoying because it interrupts the flow and then I have to keep tabbing (pressing the tab key) until I can pick up the text again I was reading.”* Participants similarly described that dismissing an ad repositioned screen reader focus to a different section of a page, causing participants to lose track of their location. Additionally, participants described automatically-played audio-video ads as unexpected noise that is disruptive of their audio-based consumption of information (P3, P4, P12, P14).

### 4.2 Interplay Among Deceptive Design Patterns

Participants additionally described interplay among deceptive design patterns, adding layers of difficulty and amplifying the complexity of resulting challenges.

‘Forced Action’ often amplified ‘Sneaking’, ‘Obstruction’, and ‘Nagging’. For ‘Sneaking’, P7 described unlabeled pre-selected checkboxes for a mailing list causing forced enrollment, saying *“I just want to buy this damn widget. And if that means [by] placing [the] order, I get signed up for the mailing list, I’ll unsubscribe later.”* For ‘Obstruction’, an unlabeled or delayed dismissal option can force unwilling engagement. P15 described how an unlabeled dismissal option in an ad on the CVS website forced engagement: *“The only way to close this ad I could find was to click on the button which took me to a page that listed all of the snack sales... this was not at all something I was looking for or wanted to do.”* For ‘Nagging’, P5 in a diary entry described a ‘Nagging’ ad on the 1440 news site advertising a ‘free’ learning service that was *“really NOT FREE”*, and that inaccessibility of this ad ultimately caused them to subscribe in order to dismiss the ad. In addition to such interplays with ‘Forced Action’, participants discussed ‘Obstruction’ commonly appearing alongside ‘Nagging’ and ‘Sneaking’. Participants often found it hard to dismiss disruptive ads (i.e., ‘Nagging’) or unintended subscriptions (i.e., Sneaking’), with P10 noting, *“Sometimes unsubscribing is harder than subscribing.”* Complexities of layered deceptive design patterns and their interplay with accessibility barriers thus produce a landscape of exacerbated challenges that can disproportionately impact people using visual accessibility technology.

### 4.3 Impacts of Deceptive Design Patterns on People Who Use Visual Accessibility Technologies

Participants described negative impacts created by the interplay between access barriers and deceptive design patterns. They also described workarounds developed to manage deceptive design patterns. Nevertheless, the tax associated with these workarounds furthers adverse consequences of deceptive design patterns.

#### 4.3.1 Financial Cost.

Participants described heightened financial costs due to deceptive design patterns, whether as a direct ramification of a pattern or as a byproduct of leveraging a workaround. Patterns like ‘Sneaking’ are problematic for all, but participants described how screen reader feedback makes them more difficult to detect. P13 reported encountering subscriptions with hidden fees, *“Getting information about what’s included in a subscription can be tricky because sometimes there may be hidden fees and it’s not always easy to tell.”* P15 shared experiences with food delivery services pre-checking higher tipping options: *“They don’t really tell you what the service charge is going to be. Sometimes they will automatically select like 20% or 25% for a tip... And so you have to go back in and look.”* ‘Misdirection’ also caused financial cost through visual styles of interface elements. P12 described how the lack of appropriate color contrast or shape distinction between buttons in the Uber app caused them to *“[end] up accidentally canceling the ride and being charged a cancellation fee”*.

As part of managing these deceptive patterns, participants (P1, P8, P9, P12, P13, P14, P15) sometimes used visual interpreting services, such as Aira [1]. However, this requires a monthly fee associated with a limited number of minutes of assistance. Considering this additional cost, participants were selective about when to employ visual assistance for navigating deceptive design. P13 shared, *“I have a small plan that I do pay for each month, but it’s only 30 minutes, so I’m judicious about what I’m going to use that for.”* P13 also expressed frustration at this additional expense due to deceptive design patterns, saying *“I don’t want to have to pay somebody to do what I should be able to do myself.”*

#### 4.3.2 Cognitive Cost.

Participants described increased cognitive effort required to complete tasks in the presence of deceptive design patterns. For example, some described getting lost due to services manipulating keyboard navigation order and redirecting screen reader focus, which then required *“more effort”* (P1, P8) to relocate the screen reader to the desired position. P14 shared, *“The way that [elements are] listed in the code is different than the way that they appear [visually]. So it’s like you’re reading along and then all of a sudden it jumps to the bottom of the page to something else, and then it jumps back up to the top.”* Low-vision participants described visual clutter from ‘Nagging’ ads made it difficult to digest desired information. P12 shared, *“If you’re going to an online store and the homepage has all those moving videos and kind of rotating ads, that is so difficult to figure out what offers or sales are going on because it won’t stop moving and I can’t read quick enough before it moves on to the next thing.”* P7 described how ‘Urgency’ induced by countdown timers on Ticketmaster required splitting attention across multiple tasks, listening to the screen reader announcing the descending countdown while inputting numbers into a text field for purchasing tickets. This became cognitively overwhelming, as P7 explained, *“If you’ve got something that’s counting backward (timers) while you’re trying to write in a credit card number, you’ve got two different series of numbers that are being thrown at you.”*

Although participants were able to devise workarounds, developing and maintaining workarounds imposed a *“learning curve,”* (P12) adding additional cognitive load. P8 described the detailed process through which they tried to navigate ‘Sneaking’ patterns caused by unlabeled elements (e.g., pre-selected checkboxes): *“I might have to route the JAWS cursor to the virtual cursor, making sure it’s right where I need it to be, and use that method of unchecking using the button that represents the left mouse button to uncheck it.”* Similarly, P7 described their attempt to navigate an ‘Obstruction’ pattern that made it difficult to edit a booked flight, *“You fight through it. It’s just like hell.”* P7 even described switching between screen readers to circumventing ‘Sneaking’, explaining *“I’m not sure if the average person would put two or three screen readers on their computer and then be expected to learn them. That’s a lot to expect someone to do.”*

#### 4.3.3 Temporal Cost.

Visual accessibility technology comes with additional temporal costs, as interfaces are often designed without consideration for accessibility [22, 31]. Participants described deceptive design patterns further magnifying temporal costs. P14 explained the *“issue of added time”*, emphasizing they must exercise greater caution: *“Whereas it might take a sighted person 20 seconds to throw in their email and name and click next, I’m probably going to double check it to make sure I actually put it in right, which might involve me going letter by letter with VoiceOver or trying to zoom in and make sure that I get all the letters in there. So it’s probably going to take me a little longer.”* P1 reported spending extra time to circumvent ‘Misdirection’ created by inaccessible visual styles or mislabeled elements: *“Sometimes the positioning of their buttons or their description... is harder to figure out and it takes a little bit of time to find out what field is associated to what description they’ve given.”* Similarly, P4 reflected on identifying ‘Sneaking’ patterns of pre-selected checkboxes, saying *“Certainly it takes more time to go through and read that option and uncheck it.”* P13 described spending additional time to avoid ‘Obstruction’ when booking a flight for clients using Alaska Airlines. Using keyboard navigation to filter for low airfare prices, it *“was harder to find located at the very bottom and did not seem to navigate well”*. Adding to challenges of temporal cost, ‘Urgency’ patterns required completing tasks within a short amount of time that participants said was not sufficient when using a screen reader. P1 stated this went against established accessibility principles: *“Not providing enough time is against the WCAG standards... I’m sure it’s a part of Section 508 [of the Rehabilitation Act] trusted testing as well. In that sense, even a lawsuit could be done by someone with a disability saying, ‘This is not accessible to me because it’s not giving me enough time.’”* Although interpreting services like Aira could help participants avoid some deceptive designs, this workaround also became *“extremely tedious and time consuming”* (P13). Overall, participants found the temporal cost of deceptive design *“frustrating”* (P14) and *“a deterrent”* (P1). P13 lamented, *“It can be really annoying if you’re working on something that’s supposed to take just a short amount of time, and then because of trying to figure out the accessibility, it takes longer.”*

#### 4.3.4 Heightened Uncertainty.

Participants described heightened uncertainty created by accessibility barriers and deceptive design patterns, often *“guessing”* (P7, P8, P9, P11, P16) their way through. For example, P7 described how they tried to make sense of unlabeled elements resulting in ‘Sneaking’ and ‘Misdirection’ patterns: *“When you’re on a webpage, if something says ‘graphic 32 button’ and you know that there ought to be a submit button, chances are pretty good that it’s going to be the ‘graphic 32 button’.”* P13 added, *“Sometimes I can guess, for instance, there might be something that says ‘username’. And then you tab and the next thing might say ‘unlabeled,’ but you can guess that it’s most likely ‘password’.”* P11 similarly adopted a trial-and-error approach for date entry when their screen reader did not announce the expected date format, re-entering dates in different formats to guess the expected format. They explained, *“The issue is there’s no standardization... As a blind person, you’re filling this thing out. You’re just guessing at the way they want it.”* P7 even resorted to speculating the intent of the developer when navigating ‘Sneaking’, *“You do a lot of guessing, and you try to get into– if I was a developer who put this thing together, what would I have done? And you try to figure out what logic, if any, they tried to use.”* Although participants occasionally succeeded in finding their way around deceptive design patterns through such speculation, they described the feeling of uncertainty it caused, equating their experience with *“playing the lottery.”* (P7). Similarly, P14 described a lack of confidence navigating uninformative image descriptions resulting in ‘Misdirection’ and ‘Sneaking’ patterns while shopping, stating *“I just didn’t feel empowered to make that decision myself because I just didn’t have full confidence in what they looked like.”*

#### 4.3.5 Decreased Agency.

Although deceptive design patterns diminish agency for everybody who encounters them, our participants described feeling more likely to be manipulated because of added accessibility barriers. For example, participants described learning or speculating the presence of a deceptive design, but then being unable to avoid it due to accessibility issues. P10 shared, *“There could be something like a check mark saying to a sighted person that this is a paid review, but my accessibility [technology] is not reading it that way”*. ‘Obstruction’ via hard-to-cancel advertisements was another example, where participants found it difficult to move past ads because of access barriers. P7 shared, *“You can’t get the pop-up to close. And the only way I found to get it to close sometimes is to close the thing out completely and reestablish the connection.”*

Many participants reported leveraging sighted assistance from family, friends, and partners. For example, P12 worked with sighted family members or volunteers from visual interpreting services to solve reCAPTCHAs, saying, *“If the audio [reCAPTCHA] is not working out, I’m going to go find a person or sometimes I might use the Be My Eyes app or send a picture of it to my mom or sister and be like, hey, who can tell me what I’m supposed to type now?”* Similarly, P12 described navigating ‘Obstruction’ of an ad interrupting in Wordle on the New York Times website, saying they *“had to pass the phone to my husband for him to close the ad.”* Although collaborative workarounds helped participants navigate deceptive designs, *“depending on someone else”* (P2) to accomplish tasks sometimes made them feel a lack of agency. P14 lamented, *“Anytime you’re relying on visual interpreting from someone else, it takes away a little bit of that power from you. And I don’t think that’s ever going to fully go away for blind people.”*

#### 4.3.6 Frustration Leading to Abandonment.

Participants described the interplay between accessibility barriers and deceptive design patterns as often leaving them unable to perform tasks that brought them to a service. P7 recalled being *“completely unable to complete the task”* on Home Depot’s website due to ‘Nagging’ subscription ads that were hard to close, and expressed feeling *“fed up”* with services employing ‘Forced Action’ to make them subscribe to undesired mailing lists. Impacts of deceptive design sometimes pushed participants away from using specific services. P5 shared where ‘Obstruction’ in a subscription pop-up ultimately prompted them switching to an alternative service: *“The close button was hard to find and not responsive...Finally I got too frustrated and navigated to a different news source.”* P12 also avoided using services when they found it difficult to *“get past”* ‘Nagging’ ads using their screen reader. They said, *“It’ll start reading like ‘video ends in 30 seconds’, ‘video ends in 30 seconds’ and it gets on a stuck loop. So when that happens, I might have to go to a new website.”* To address such ‘Nagging’, P6, P13, and P15 described trying ad blockers, but abandoned those, because they *“were blocking some things that I actually cared about... [and] certain things that were really hard to do with the ad blockers in place”* (P15). Thus, when workarounds were not effective against *“annoying”* (P14, P8) deceptive design patterns, participants reported abandoning tasks momentarily or altogether. P15 said, *“Once in a while, if I don’t care that much, I’ll stop. I’ll just say it’s not worth it.”*

#### 4.3.7 Ableism and the “Backfiring” of Deceptive Design.

Expanding upon their discussion of abandoning deceptive and inaccessible services, participants further discussed decisions to abandon as contrary to presumed intent for including deceptive patterns. For example, services deploy countdown timers to accelerate decision-making through a sense of ‘Urgency’. However, participants described how their screen reader announcement of the descending time pulled attention away from the task, thus making it require extra time to complete or even leading to abandonment. P13 noted, *“If it’s going to have things that are counting down timers, I’m going to be less likely to use [it].”* The interplay between deceptive design and access barriers can thus result in reduced engagement, contrary to the motivation of deceptive design in isolation. P4 described how a ‘Nagging’ ad failed to achieve its presumed intent because an automatically spinning carousel induced motion sickness, *“Joke’s on them, you make me want to look at your advertisements less when you make them move. So maybe that’s like an anti-dark pattern where the choices you’re making are making me less likely to view your advertisements.”* Deceptive designs that aimed to manipulate people into purchases also deterred participants due to associated access barriers as well as privacy and security concerns related to workarounds (e.g., sighted assistance). P1 shared, *“If it’s a payment page, if it’s not accessible, I probably wouldn’t make the purchase from that website at all, because I don’t really want to share payment details or any sort of confidential information of that kind, even with a trusted service like Aira. Because I think I need to be careful.”*

Such participant experiences further highlight that accessibility is often not a primary concern, including among developers who intentionally implement deceptive design patterns. Accessibility barriers caused by these deceptive designs were severe enough that participants abandoned the interaction, with the deceptive design thus effectively “backfiring” (P14). P13 also noted that using a screen reader inherently changes interaction with a deceptive design, saying *“I may be missing some dark patterns. I may be skipping over some of the stuff, which could be an advantage.”* Participants perspectives overall highlight the embedded ableism within established deceptive patterns, including contexts in which developer assumptions are counterproductive.

## 5 Discussion

### 5.1 Intent versus Impact of Deceptive Design Patterns

Poor design can result from a designer’s flawed assumptions, competing priorities, limited knowledge, or failure in implementation. Although many accessibility barriers result from poor design, our results highlight the interplay between accessibility barriers and deceptive design, wherein deceptive patterns cause disproportionate direct and indirect harms to people who use visual accessibility technology. We originally intended a focus on accessibility and additional harms in *intentional* instances of deceptive design patterns, but participants additionally described examples where they felt accessibility barriers resulted in deceptive designs that only manifested via visual accessibility technology. We cannot be certain of any designer’s intent, but it seems likely such examples of deceptive design patterns are a result of poor design. De-prioritization or a lack of attention to accessibility thus creates designs that can be *unintentionally* deceptive, subverting decision-making while directly and indirectly inflicting harms.

Much like modern regulation does not allow poor design as an excuse for poor accessibility (i.e., regulations require accessibility regardless of factors like a designer’s flawed assumptions or competing priorities), modern regulatory definitions of deceptive design patterns do not require intent in deception, but rather focus on the impact of design decisions or design inaction (e.g., the European Union’s Digital Services Act is explicit that deceptive patterns may be *“either on purpose or in effect”*2; regulation enacting the California Privacy Rights Act is similarly explicit that *“intent in designing the interface is not determinative”*3). This is consistent with Section 4’s reporting of participant descriptions of their experiences, as participants emphasized both accessibility in intentionally deceptive designs and experiences with accessibility barriers manifesting as deceptive. It also suggests opportunities for alignment of interests in research, practice, and regulation. Particularly in contexts where potential harms are more understood and regulated (e.g., unsubscribing from a paid service, unsubscribing from email lists, properly labeling advertisements), our results highlight that inattention to accessibility is effectively deceptive and should not be dismissed as simple poor design.

### 5.2 Harms of Deceptive Design Patterns for People Who Use Visual Accessibility Technologies

**Figure 4:**
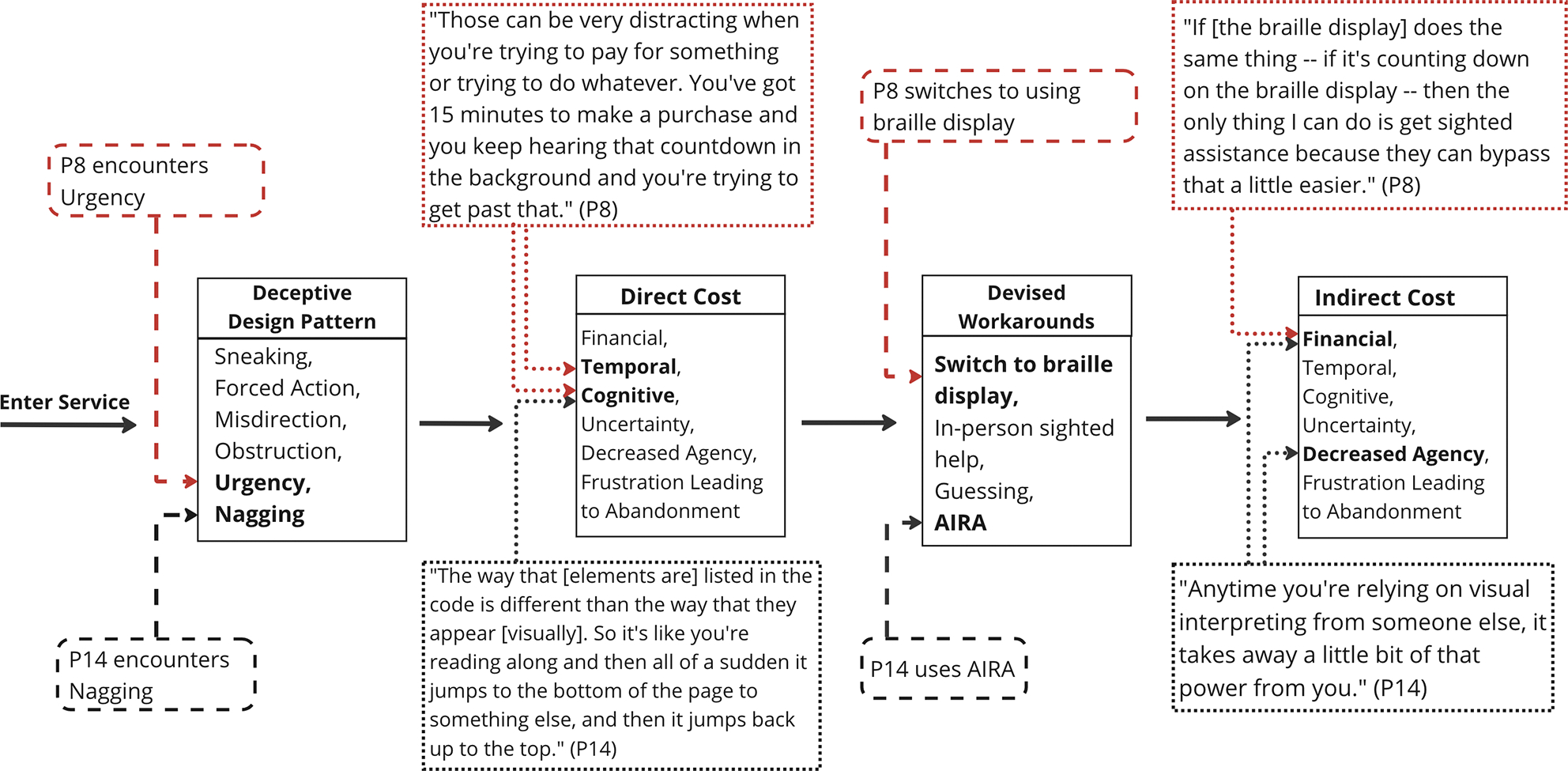
An illustration of stages of direct and indirect costs described by participants in their experiences with the interplay between deceptive design patterns and accessibility barriers. A linear progression emphasizes continuous taxes people who use visual accessibility technology must navigate, including direct costs of a deceptive design and indirect costs of workarounds.

Participant experiences and our results are consistent with prior analyses of experiences with deceptive design patterns [9, 33, 52, 54, 58, 60], while also highlighting the interplay with access barriers to create disproportionate negative impacts for people who use visual accessibility technology. For example, Mathur et al. analyzed prior deceptive design literature in attempt to answer: *“What, exactly, makes a user interface design a dark pattern?”* They developed a taxonomy identifying individual and collective welfare as two high-level concerns of deceptive design, further characterizing individual harms in terms of financial loss, invasion of privacy, and cognitive burden [58]. Informed by our results, we revisit participant-reported consequences of Section 4.3 through Mathur et al.’s lens of individual welfare. We extend Mathur et al.’s prior results through further characterizing financial and cognitive costs, through participant descriptions of emotional costs not previously characterized by Mathur et al., and through characterization of direct versus indirect costs across these categories and how participant-reported workarounds uniquely contribute to indirect harms as a result of employing them to navigate the interplay with accessibility barriers (see Figure 4).

#### 5.2.1 Financial Costs.

Mathur et al.’s taxonomy of harm [58] identifies “financial loss” as an individual harm. Participants described being more likely to incur financial costs when encountering deceptive design patterns due to their interplay with accessibility barriers. For example, a ‘Sneaking’ pattern with hidden costs may not be revealed through spoken feedback due to unlabeled or uninformative elements. Participants expressed that added accessibility barriers made it more difficult to uncover hidden costs, leading to amplified direct financial consequences.

Participants further described indirect financial costs associated with workarounds to navigate inaccessible designs. Many used visual interpretation services like Aira to achieve their access needs. Although this co-creation of access [4] offers benefit, obtaining virtual sighted help also imposes financial cost. Financial costs associated with deceptive design thus include both direct costs and these additional indirect costs associated with workarounds.

#### 5.2.2 Cognitive Costs.

Mathur et al.’s taxonomy of harm [58] further identifies “cognitive burden” as an individual harm, describing expenditure of “unnecessary time, energy, and attention”. This notion of cognitive burden was prevalent for participants (see Sections 4.3.2 and 4.3.3). Navigating online services with accessibility barriers has cognitive and temporal costs, but participants further described those costs as magnified in their interplay with deceptive design.

‘Obstruction’ patterns caused difficulty with dismissing popup advertisements (e.g., due to unlabeled dismissal elements), requiring additional time and effort to determine how to return to the intended task. Beyond spending time attempting to close an ad, participants described needing to refresh a page, recruiting sighted assistance, or being forced to engage with an unwanted ad (i.e., all further increasing cognitive costs, and further producing a ‘Forced Action’ pattern). Manipulated tab order also amplified time and cognitive costs: dismissing an advertisement sometimes changed a screen reader’s cursor positioning, requiring more effort to reposition it in resuming a task. In other cases, a desired ‘Unsubscribe’ button was located at the bottom of a page, requiring tabbing throughout the entire page. In ‘Urgency’, pressure to accelerate decision-making caused by countdown timers is accompanied with additional cognitive cost created by distracting announcement of the countdown.

Participant workarounds resulted in their own indirect cognitive and temporal costs. For example, utilizing Aira required additional time to obtain sighted help and felt tedious to some. Participants described a sometimes complex labor in assessing whether employing strategies to acquire access is worth the potential ramifications. The extra time and effort spent performing this complex calculus [53] thus increases cognitive and temporal costs.

#### 5.2.3 Emotional Costs.

Participants described emotional costs as an additional harm not captured by Mathur et al.’s taxonomy of harm. Chordia et al. recently proposed an expansion to individual welfare to surface “emotional load” [18]. They document a need to explore this harm, but their analysis only points towards fear. We identify three additional concrete emotional costs and further examine how these costs impact participants: emotional costs of heightened uncertainty (Section 4.3.4), decreased agency (Section 4.3.5), and frustration (Section 4.3.6). These advance understanding of individual harms of deceptive design patterns, and we additionally explore indirect emotional costs of participant workarounds, which can be *“exhausting”* (P12).

Emotional cost of uncertainty often accompanied navigating an inaccessible service, which was exacerbated by deceptive design patterns and their additional accessibility barriers. Participants described navigating deceptive designs as a matter of guessing. Participant workarounds included engaging social support networks to co-create access, but this brought its own emotional costs, with an individual weighing their access needs against uncertain social costs of feeling a burden to others [23]. Uncertainty around these multiple potential costs can thus shape whether people seek help in navigating a deceptive design.

Deceptive design patterns inherently compromise agency in manipulating and steering people to undesirable actions [21, 58], but participants described interplay with access barriers as amplifying this impact. Access barriers eliminated opportunities for participants to discover the presence of deceptive patterns, which in turn diminished their opportunity to object. In cases where participants were aware of a deceptive design, associated access barriers preserved a pattern’s obstructive nature, making it difficult to avoid. A feeling of decreased agency carried into participant workarounds, with some describing a decreased sense of agency when needing to obtain sighted help.

When encountering deceptive design, participants discussed difficulty performing the desired tasks for which they originally came to a service. Participants described frustration and abandonment of services, as accessibility barriers and their interplay with deceptive design presented unnecessary difficulty (Section 4.3.6). Workarounds of requesting sighted help or using visual interpretation services were also frustrating for some participants, because they felt they should be able to complete tasks on their own. When a workaround was not effective in managing a deceptive design, participants described giving up because it was not worth the frustration.

#### 5.2.4 Direct versus Indirect Harms.

Direct harms resulting from the interplay between accessibility barriers and deceptive design patterns manifest across financial, cognitive, and emotional dimensions (e.g., as summarized in Table 3). Financial costs can emerge when people who use visual accessibility technology encounter unlabeled or uninformative promotional deals and pre-checked boxes with additional fees, leading to unintended expenses. Cognitive costs can incur through the increased time and mental effort required to navigate manipulated tab orders, unlabeled or uninformative dismissal options, delayed dismissal options, and the cognitive overload induced by disruptive countdown timers. Emotional costs can stem from the uncertainty associated with having to guess one’s way through inaccessible and deceptive design patterns, frustration due to unnecessary difficulties in completing tasks, and that frustration culminating in abandonment. Additionally, this interplay can result in experiencing decreased agency due to accessibility barriers creating challenges in detecting deceptive patterns, thereby diminishing the opportunity to object.

In addition to direct harms, we reveal a range of indirect harms resulting from the interplay between accessibility barriers and deceptive design patterns that further exacerbate challenges experienced by people who use visual accessibility technology (e.g., also summarized in Table 3). Indirect harms extend beyond immediate consequences and emerge from employing workarounds to navigate and manage compounded challenges of this interplay. Indirect harms leads to financial, cognitive, and emotional costs of deceptive designs that are unique to using visual accessibility technology. Indirect financial costs arise via fees associated with using services like Aira to obtain sighted help. Indirect cognitive costs include the increased time and effort to acquire sighted help, weighing trade-offs between attempting to navigate deceptive design themselves versus additional accessibility barriers these patterns might introduce, assessing social costs of asking for sighted help, switching between accessibility technologies to find the most effective means of access, and the increased time spent navigating the interplay when sighted help is unavailable due to privacy concerns. Indirect emotional costs can include uncertainty in acquiring sighted help, heightened sense of decreased agency when relying on sighted help, and frustration associated with obtaining sighted help.

### 5.3 Deceptive Design Patterns, Tradeoffs, and Consequence-Based Accessibility

In discussing workarounds for navigating deceptive design patterns, many participants described being forced to choose between multiple negative experiences, rather than being able to identify a reliable solution. These often involved tradeoffs, requiring they decide what parts of an experience were most important or what negative experiences they were willing to tolerate. For example, some participants described using an ad blocker to manage hard to close ads in ‘Obstruction’ patterns, but with a tradeoff that the ad blocker alters page functionality and introduces a new set of access barriers. Challenges of ad blockers introducing new accessibility barriers have also been reported by Kodandaram et al. [47]. Participants additionally described a tradeoff in paying a monthly subscription for Aira to avoid ‘Sneaking’ and to be confident in their desired tasks.

In engaging these tradeoffs, participants were forced to choose between ease of use, confidence in their ability to complete a task, and expected level of precision or correctness. Consequences participants accepted in these tradeoffs correspond to those in Section 4.3: additional financial costs, the inability to use certain features of a service, a higher time and cognitive cost, and negative emotional experiences involving frustration, uncertainty, and decreased agency.

Tradeoffs are a long-established phenomenon in interface design [64], but are less documented in how people make decisions around interacting with a system, particularly with regard to accessibility. We can understand this manifestation of tradeoffs as an extension of Mack and McDonnell et al.’s framework of *consequence-based accessibility* [53]. Rather than viewing accessibility as a binary *accessible* or *inaccessible*, participant experiences demonstrate how online accessibility often involves personal calculations of what is *accessible enough*. This also shows participants being forced to ask what accessibility is worth to them in a particular context, including a consideration of what consequences they are willing to face. This invisible complex labor [4, 12, 78] of assessment also contributes additional cognitive and temporal costs. Our research thus further highlights importance of the consequence-based accessibility framework for informing accessible design across diverse ranges of abilities, extending the framework from Mack and McDonnell et al.’s original focus on a chronically ill population to also characterize experiences of people using visual accessibility technology (i.e., highlighting a similar need for understanding accessibility-related tradeoffs and consequences, while characterizing such tradeoffs and consequences within our different focus).

### 5.4 Design Considerations for Accessibility Tools

Deceptive design patterns are inherently negative for people who encounter them, but our results highlight that deceptive design can be unintentional with amplified negative consequences for people who use visual accessibility technology. It is first and foremost clear that designers should not intentionally produce deceptive designs. Addressing the interplay between accessibility and deceptive design therefore can begin with non-technical measures, wherein well-intentioned designers enhance their education and literacy around deceptive design patterns and their interplay with accessibility practices and standards. By prioritizing these competencies and their integration throughout the design process, designers cultivate a critical awareness that supports proactively addressing potential barriers before they emerge.

Our identification of the potential for unintentional deception also suggests improving accessibility tools to support well-intentioned designers. Automated accessibility testing tools are limited in providing adequate evaluations to improve accessibility [56, 73]. One of these limitations is that automated tools generally identify low-level accessibility failures (e.g., unlabeled buttons) and provide feedback on how to solve the problem. Although useful, testing tools generally stop there, not providing supplemental context on how a failure will negatively impact people or why it is important to address the failure. Because not all low-level failures are the same (e.g., some result in deceptive designs that have direct and indirect costs), accessibility tools have an opportunity to go beyond low-level warnings to also call out higher-level implications and priorities. For example, an accessibility checker might identify an unlabeled checkbox and flag that low-level failure. Informed by our results, such an accessibility checker might further identify that an unlabeled pre-selected checkbox controls addition of an shipping insurance fee to a shopping cart and therefore additionally flag the higher-level ‘Sneaking’ pattern created by this accessibility failure. Automated testing is just one tool to support designers alongside other methods (e.g., usability testing, expert evaluations, community feedback), but such higher-level automated feedback might help keep designers more aware of potential impacts of access barriers and inform prioritization in their repair.

## 6 Limitations and Future Work

We largely examine experiences of people who use screen readers while encountering deceptive design patterns, many of whom identify as blind or low-vision. Six participants reported using other visual accessibility technologies apart from screen readers (e.g., screen magnification, braille displays, voice assistants). Due to the varying functionalities of accessibility technologies, deceptive design patterns may manifest differently across them. Future research should explore in greater detail how the effects of deceptive design patterns vary across accessibility technologies.

Our review of online services in which participants reported deceptive design patterns was sometimes limited (e.g., due to changes in those services preventing inspection of a design), leaving some susceptibility to recall bias and misinterpretation. Our diary study was also not designed to elicit all encounters with deceptive design patterns, so our data does not support conclusions about their prevalence or the frequency with which people encounter deceptive design patterns. Finally, our method focused on experiences with deceptive design patterns, without explicitly differentiating examples that were likely intentionally deceptive versus examples that manifested due to likely unintentional accessibility barriers. Given our results highlighting these different manifestations of deceptive design patterns, future research could more explicitly explore approaches to both.

Our examination of the impacts of the interplay between deceptive design patterns and accessibility barriers with people who use visual accessibility technology also motivates consideration of compounded consequences for people across other disability groups. Considering the high prevalence of accessibility barriers, future research should explore this interplay more broadly, accounting for the experiences of people across disabilities, chronic health conditions, nuerodivergency, and mental health conditions. As discussed by Chordia et al., there is a potential for deceptive design patterns to interact with cultural and social contexts to systematically reproduce negative stereotypes and reinforce cultural biases [18]. For example, future research should examine how deceptive patterns can further exacerbate consequences for people of color with disabilities when using services (e.g., healthcare, housing, governmental) that perpetuate the historical continuum of discrimination against these communities, thus aiming to better understand and address accessibility in terms of such intersectional experiences [43].

## 7 Conclusion

Our investigation of the interplay between accessibility barriers and deceptive design patterns reveals both exacerbated impacts of deceptive design on people who use visual accessibility technology and how people who use visual accessibility technology describe feeling that accessibility barriers manifest as deceptive designs. In an interview and diary study with 16 people who use visual accessibility technology, participants described experiences with six deceptive design patterns: ‘Sneaking’, ‘Forced Action’, ‘Misdirection’, ‘Obstruction’, ‘Urgency’, and ‘Nagging’. Participants described direct and indirect harms in the interplay of accessibility and these deceptive design patterns. Participant experiences demonstrate the impact of deceptive designs regardless of designer intent. Our work extends existing understanding of financial and cognitive costs of deceptive design, both by illustrating direct and indirect implications and by identifying additional direct and indirect emotional costs. We additionally consider these harms from a perspective of consequence-based accessibility, extending this framework to include people who use visual accessibility technology. We finally outline an opportunity for accessibility tools to account for deceptive design patterns in providing higher-level feedback to well-intentioned designers. Our work thus provides new understanding and insight into both accessibility and deceptive design through our examination of their interplay across a wide variety of participant experiences with visual accessibility technology.

## Figures and Tables

**Table 1:** Overview of self-reported participant information, accessibility technology, years of experience using accessibility technology, and types of services accessed using that technology. 15 participants reported using screen readers, and many reported using additional visual accessibility technology.

ID	Reported Gender	Reported Disability	Reported Accessibility Technology	Reported Years of Experience	Reported Types of Services
P1	Male	Blind	JAWS, Voiceover	15 years	Banking, Entertainment, Shopping, Travel
P2	Male	Blind	Google Assistant, Google Home	3 years	News and Weather
P3	Male	Low-Vision	NVDA	1 year	Banking, Education, Shopping
P4	Genderqueer	Chronically ill	Narrator, NaturalReader	4 years	Blogs, Education, Shopping, Social Media
P5	Female	Legally blind in one eye, end-stage glaucoma	Magnification, high contrast, inverted colors, screen reader	30+ years	Banking, Entertainment, Medical, Shopping, Travel, Weather
P6	Male	Blind	JAWS, Voiceover, Orbit Reader braille display	35+ years	Banking, Medical, Shopping
P7	Male	Blind	JAWS, NVDA, VoiceOver, TalkBack, braille displays (i.e., Focus, Brailliant, QBraille)	33 years	Banking, Medical, Shopping
P8	Female	Blind	JAWS, braille displays (i.e., Focus Blue, HandiTech Active Braille)	28 years	Banking, Shopping
P9	Male	Blind	JAWS and Voiceover	10+ years	Banking, Entertainment, Shopping, Social Media
P10	Female	Blind	Voiceover	12 years	Banking, Shopping, Travel
P11	Male	Blind with light perception	JAWS, Voiceover	23 years	Banking, Shopping
P12	Female	Blind in left eye, limited vision in right eye	Zoomtext, Voiceover	12 years	Education, Email, Shopping, Social Media
P13	Female	Blind with light perception	JAWS, NVDA, Talkback, Voiceover, braille display	30+ years	Blogs, Shopping, Social Media, Medical
P14	Female	Blind	Voiceover, JAWS, Magnification	12 years	Banking, Shopping, Social Media, Travel
P15	Female	Blind	JAWS, Voiceover, Brailliant braille display	25 years	Banking, Shopping
P16	Female	Macular degeneration and severe astigmatism	Fusion	35+ years	Banking, Shopping

**Table 2:** Participants reported experiences with six deceptive design patterns, describing how the interplay between these deceptive design patterns and associated access barriers created additional challenges and consequences.

Category	Description	Related Access Barriers	Participant Example
Sneaking [35, 57]	Misrepresenting actions by hiding or delaying information	Unlabeled and uninformative interface elements	An unlabeled pre-selected checkbox to sign up for a promotional plan or mailing list (P13,P15)
Forced Action [35, 57]	Require additional tangential actions to continue in a desired task	Inaccessible authentication systems	A reCAPTCHA that requires performing an inaccessible audio recognition task (P3, P6, P7, P9, P16)
Misdirection [57]	Use presentation, language, and emotion to steer people toward or away from certain decisions	Inaccessible stylistic elements	A ‘Cancel’ or ‘No Thanks’ button with low color contrast, small text size, and/or illegible font style (P12, P13)
Obstruction [35, 57]	Making actions needlessly difficult in order to dissuade those actions	Unlabeled and uninformative interface elements	An unlabeled dismissal option or unsubscribe link (P5, P8, P10, P13, P14) Unsubscribe link is placed lower on keyboard navigation order (P4)
Urgency [57]	Impose deadlines or time constraints to accelerate decision-making and action	Disruptive countdown timers	A screen reader continuously announcing the descending time on a countdown timer (P1, P4, P7, P8, P13, P14)
Nagging [35]	Repeated intrusion of unexpected functionality unrelated to a person’s task	Unlabeled and disruptive advertisement overlays	A pop-up ad that diverts screen reader focus and/or is placed higher up inkeyboard navigation order (P3, P4, P12, P14)

**Table 3:** Overview of direct and indirect harms and associated costs described by people who use visual accessibility technology. Harms result not only from accessibility barriers, but also their interplay with deceptive design patterns and workarounds.

Harms of Deceptive Design Patterns			
Direct Harms		Indirect Harms	
Financial	Unlabelled or uninformative pre-checked boxes with additional fees or promotional deals (P1, P4, P10, P14, P16) Low color contrast leading to expenses (P13)	Financial	Fees for sighted help services (e.g., Aira) (P1, P8, P9, P12, P13, P14, P15)
Cognitive	Navigating manipulated tab order for ads and unsubscribe options (P1, P3, P4) Navigating unlabelled or uninformative dismissal options (P7, P9, P10, P11, P13, P14, P15) Navigating delayed dismissal options (P4, P7, P10, P14, P15) Disruptive countdown timers (P7, P8, P10, P13) Disruptive ads (P2, P3, P4, P5, P7, P9, P11) Navigating unlabelled or uninformative check boxes (P1, P4, P10, P14, P16)	Cognitive	Added time and effort to acquire sighted help (P2, P4, P13, P15) Weighing trade-offs (P6, P12, P13, P15) Weighing social costs (P13) Guessing (P4, P5, P7, P9, P11, P13, P15) Switching to alternative AT (P8) Conflicts with sighted help (i.e., unavailable, privacy concerns) (P1, P3, P15) Restarting service to remove disruptive ads (P7, P11, P12, P14, P16)
Emotional	Uncertainty due to navigating interplay (P1, P6, P7, P11, P13, P14) Frustration due to difficulty performing the desired tasks (P5, P7, P15) Inaccessibility decreasing agency in detecting and objecting patterns (P7, P10)	Emotional	Uncertainty in acquiring sighted help (P1, P13) Decreased agency from acquiring sighted help (P2, P12, P14) Frustration from acquiring sighted help (P13)

## CCS Concepts

• Human-centered computing → Accessibility
